# Correction to “Manganese Catalyzed Dehydrogenative Synthesis of Urea Derivatives and Polyureas”

**DOI:** 10.1021/acscatal.3c02871

**Published:** 2023-08-02

**Authors:** Aniekan Ekpenyong Owen, Annika Preiss, Angus McLuskie, Chang Gao, Gavin Peters, Michael Bühl, Amit Kumar

The reaction scheme of Table 1 should be the following:
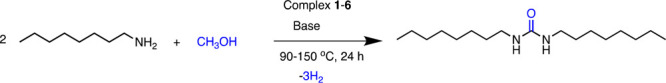


Additionally, it has come to our attention that during evaluation of the DFT results the MHP “pressure correction” was wrongly applied in several instances. While the Δ*E* and Δ*H* values included in the figures and schemes are unaffected by this, a few of the reported Δ*G* values are off in the range of ±5.4 kcal/mol. The corrected Figures 4 and 5 from the paper are presented here; the corrected schemes from the original SI are provided as new Supporting Information.

**Figure 4:**
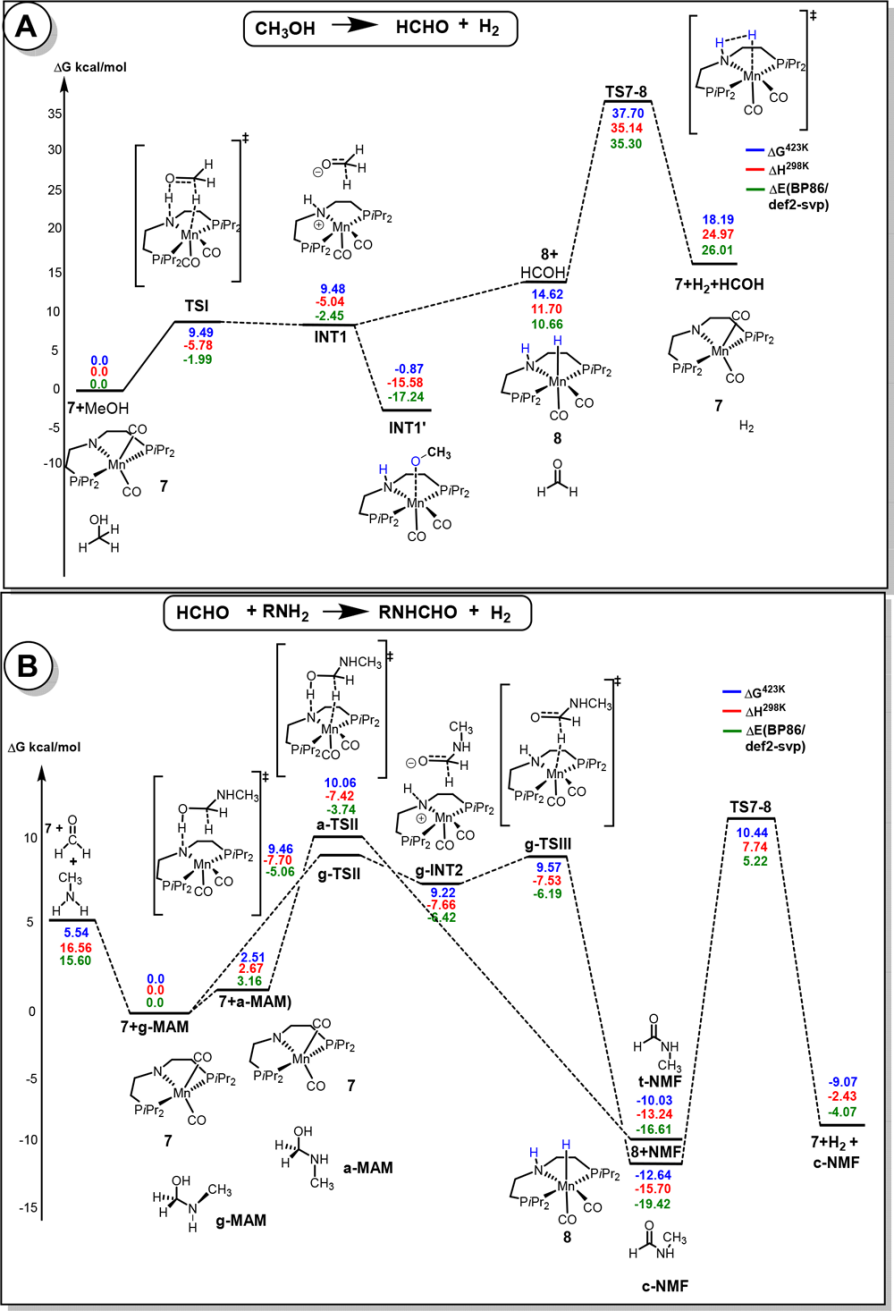
Free energy profile for (A) dehydrogenation of methanol using catalyst **7** to give formaldehyde and (B) synthesis of formamides from the dehydrogenative coupling of amines and methanol (using methylamine as a model substrate, PBE0-D3/def2-TZVP/PCM//RI-BP86/def2-SVP/PCM level).

**Figure 5:**
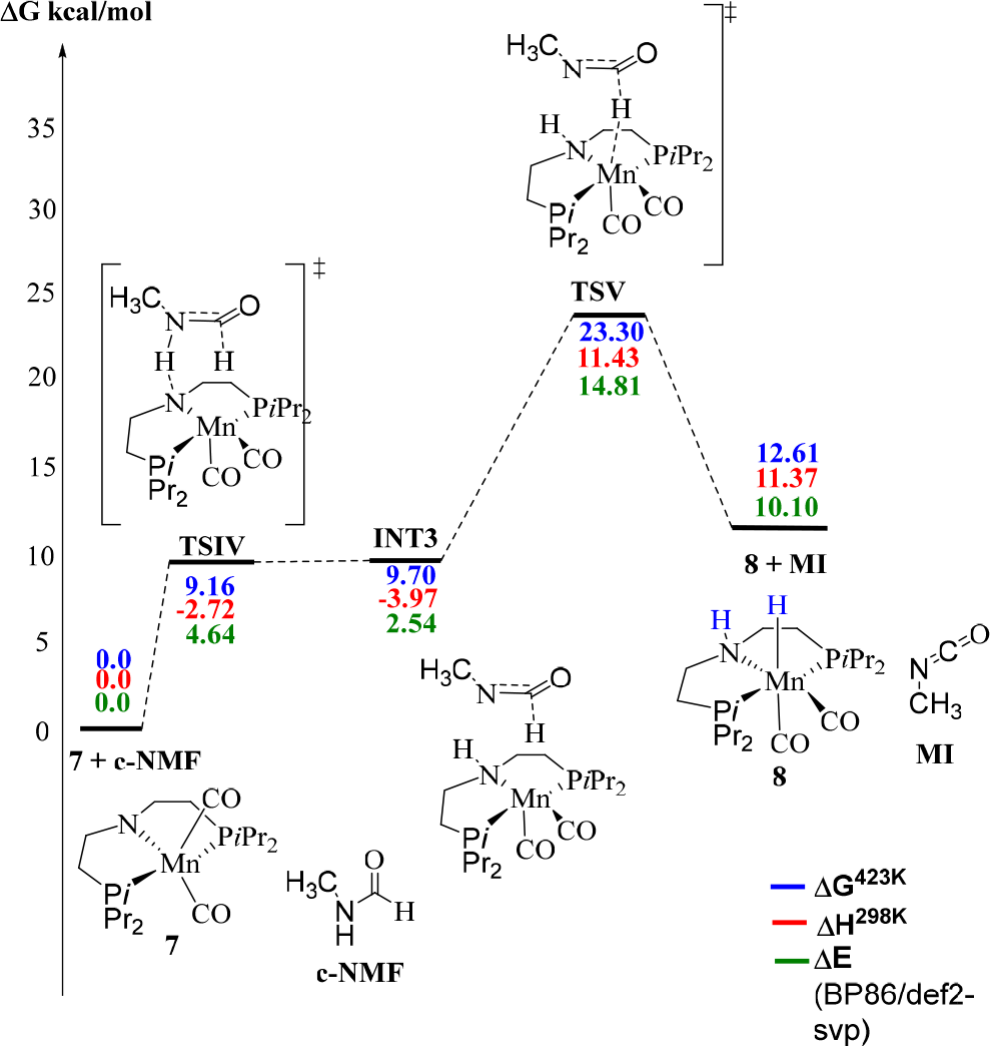
Key steps for isocyanate formation with the active catalyst **7** from DFT (using *cis*-*N*-methylformamide, **c-NMF**, and methyl isocyanide, **MI**, as model substrates, PBE0-D3/def2-TZVP/PCM//RI-BP86/def2-SVP/PCM level).

Page 6930, end of paragraph above “Step 2”: This changes some of the numerical values discussed in the text, most notably the overall barrier for catalyst regeneration. “On the profile in Figure 4A, **INT1′** is an off-cycle intermediate that has to revert back to **INT1** for the reaction to proceed, raising the rate-determining barrier to Δ*G*^‡^ = 38.6 kcal/mol between **TS7-8** and **INT1′**” (rather than 33.9 kcal/mol as reported initially). As discussed elsewhere in the paper, this barrier is reduced through participation of a solvent MeOH molecule by 5.4 kcal/mol (see Scheme S6 in the original SI), resulting in an overall barrier that is still compatible with the experimental conditions (high temperature).

Page 6930, end of paragraph above “Step 3”: “For synthesis of formamide from the dehydrogenative coupling of formaldehyde and amine (Figure 4B), the rate-limiting step is again indicated to be the regeneration of the active catalyst **7** from **8**, but now with an overall barrier Δ*G*^‡^ of only 23.1 kcal/mol between **TS7-8** and **8+c-NMF**” (instead of 18.0 kcal/mol reported initially).

Page 6930, bottom of second column: “Dehydrogenation of formamide affording methyl isocyanate is predicted to proceed via a zwitterionic intermediate akin to that involved in methanol dehydrogenation (labeled **TSI** in Figure 4A), namely, **INT3** in Figure 5, and a transition state (**TSV**) with a moderately high barrier of Δ*G*^‡^ = 23.3 kcal/mol (Figure 5). As in the Mn-catalyzed dehydrogenation steps discussed above, regeneration of the active catalyst **7** from **8** is indicated to be rate-limiting with an overall barrier of Δ*G*^‡^ = 35.7 kcal/mol; see full profile in Scheme S2)” (rather than 30.6 kcal/mol reported initially). “This barrier is similar to (and even slightly lower than) that for methanol dehydrogenation. Again, assistance by MeOH is computed to reduce both barriers by the same amount, 5.4 kcal/mol.”

Page 6931, paragraph before Conclusions: “In contrast, a higher overall barrier is computed for the aminal route (*pathway a* of Scheme 2). While catalytic dehydrogenation of a model aminal is indicated to be kinetically feasible at our DFT level, the formation of such an aminal from the formamide and alkylamine is so unfavorable (computed Δ*G* = 20.6 kcal/mol for the methylated models) that the overall barrier is raised to Δ*G*^‡^ = 33.2 kcal/mol; cf. the difference between **7+c-NMF** and **gg-TSV**I (see Schemes S4 and S5)” (instead of 38.3 kcal/mol reported initially). “At first glance this value seems lower than that computed for the isocyanate route (Δ*G*^‡^ = 35.7 kcal/mol, see above); however, while the latter is predicted to be reduced by 5.4 kcal/mol through MeOH assistance, no such solvent assistance is expected for the former, suggesting that it is the isocyanate pathway that is followed predominantly, in full accord with the experiment.”

In summary, none of the conclusions from our original study is affected by these changes.

## Data Availability

The research data supporting this publication can be accessed at 10.17630/a924bc5f-7d77-4372-b0c3-69d02ef1d090.

## Supporting Information Available

Corrected Schemes S2, S4, S5, and S7 (PDF)

