# Supplementary material for: Correction to “Manganese Catalyzed Dehydrogenative Synthesis of Urea Derivatives and Polyureas”
Source: ACS Catal. 2023 Aug 2;13(16):10796–7. doi: 10.1021/acscatal.3c02871 (PMC10442911; doi:10.1021/acscatal.3c02871)
Supplement: Supplementary file 1 — cs3c02871_si_001.pdf [file cs3c02871_si_001.pdf]

Correction to "Manganese Catalysed Dehydrogenative Synthesis of Urea Derivatives and Poly-ureas"

Aniekan Ekpenyong Owen, Annika Preiss,† Angus McLuskie,† Chang Gao,† Gavin Peters, Michael Bühl,* Amit Kumar*

ACS Catal. **2022**, *12*, 6923–6933. DOI: 10.1021/acscatal.2c00850.

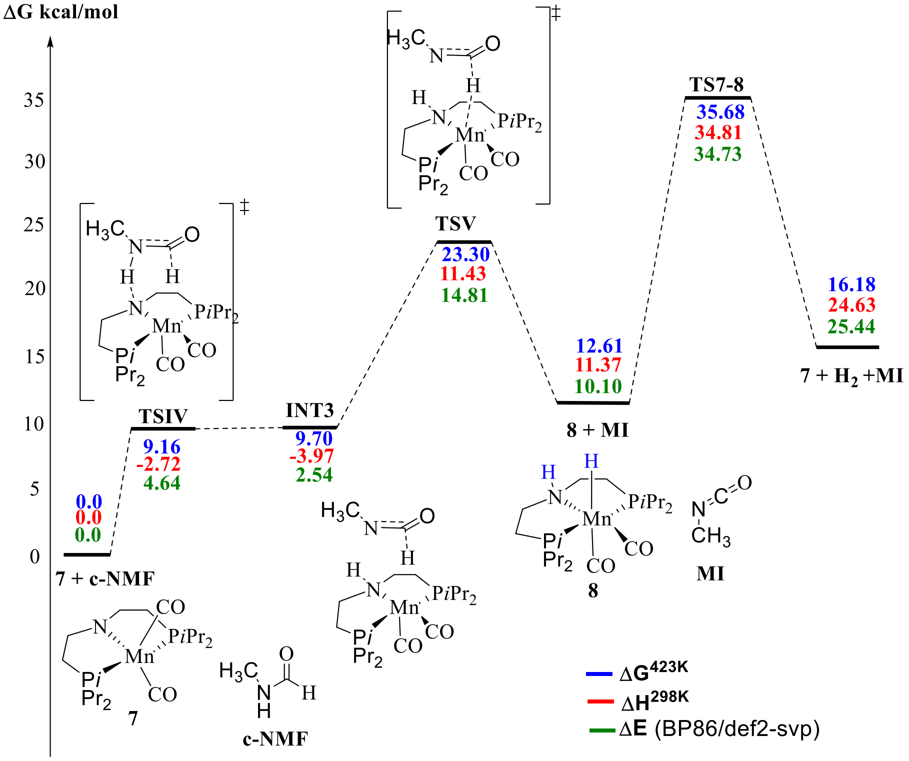

Scheme S2. Free energy profile for isocyanate formation with the active catalyst **7** (using cis-N-methylformaide, **c-NMF**, and methyl isocyanide, **MI**, as model substrates), at the PBE0-D3[pcm,THF]/def2-TZVP//RI-BP86[pcm,THF]/def2-SVP level of theory.

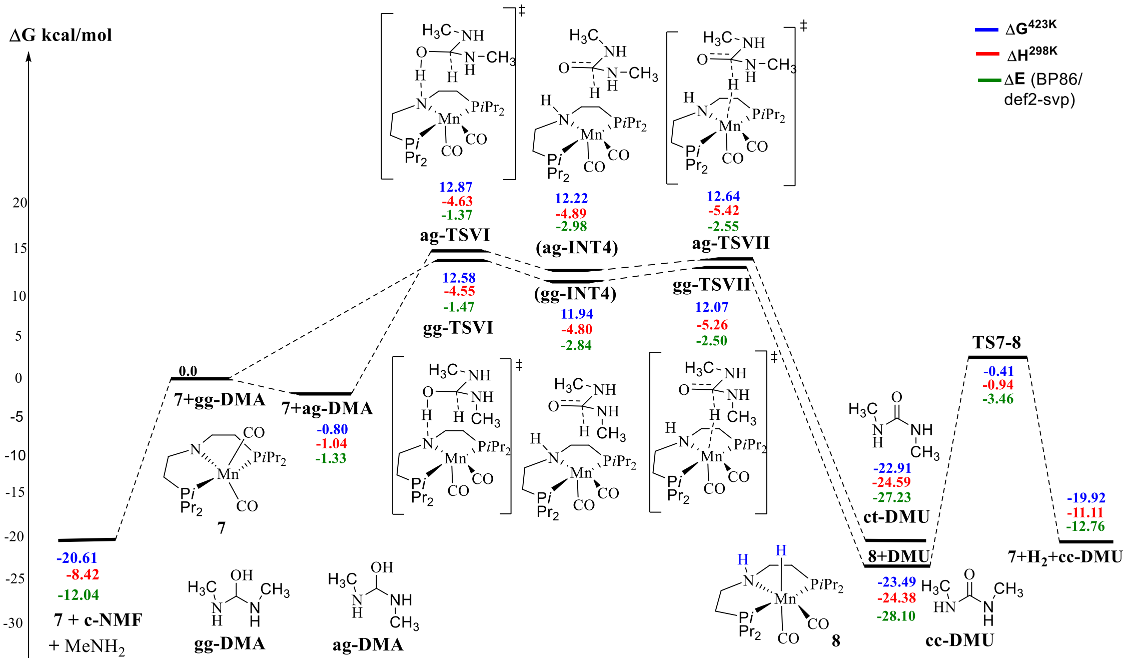

Scheme S4. Free energy profiles for the proposed pathways for dehydrogenative coupling of N-methylformamide (**NMF**) with methylamine (**MAM**) using catalyst **7** to give N,N'-dimethylurea (**DMU**) at the PBE0-D3[pcm,THF]/def2-TZVP//RI-BP86[pcm,THF]/def2-SVP level of theory.

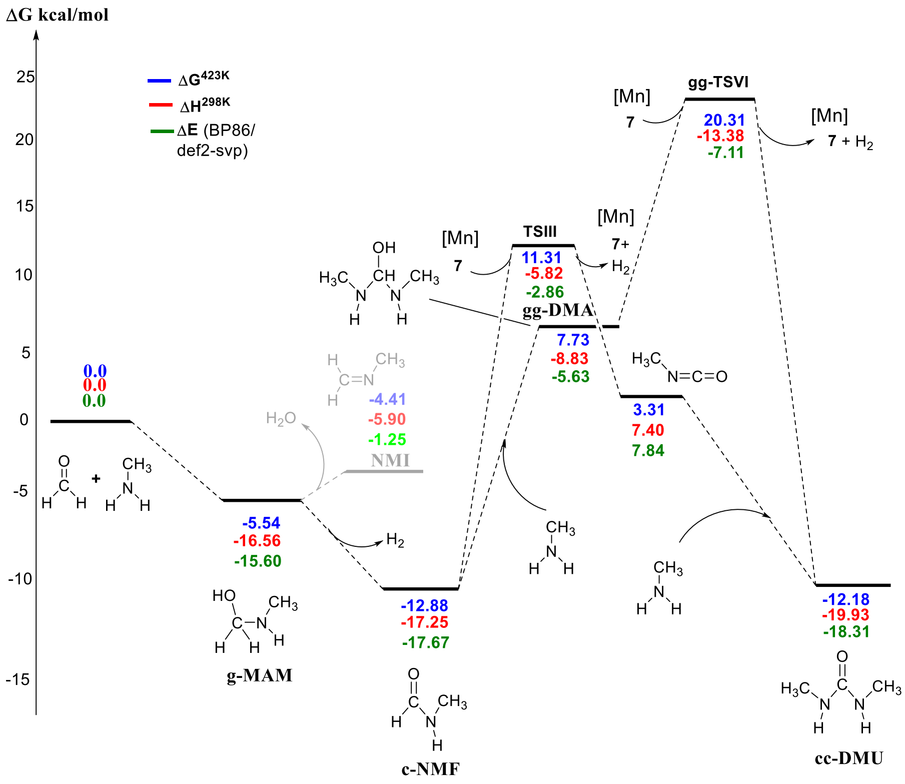

Scheme S5. Thermodynamic driving forces for formation of urea and key barriers for Mn-catalyzed aminal and isocyanate routes at the PBE0-D3[pcm,THF]/def2-TZVP//RI-BP86[pcm,THF]/def2-SVP level of theory.

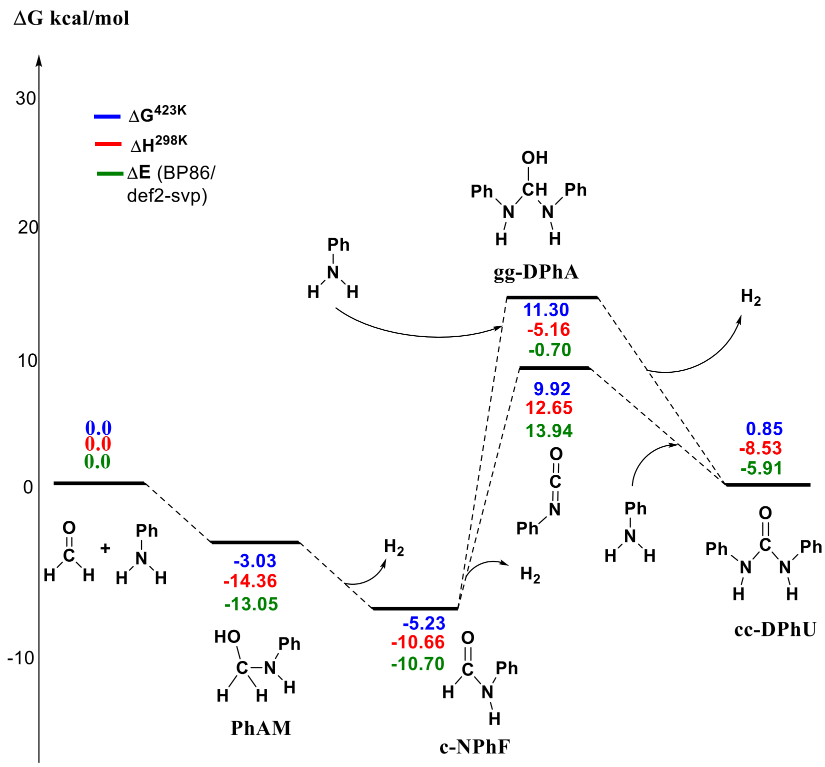

Scheme S7: Thermodynamic driving forces for formation of urea using aniline as substrate at the PBE0-D3[pcm,THF]/def2-TZVP//RI-BP86[pcm,THF]/def2-SVP level of theory.
